# High-throughput exploration of activity and stability for identifying photoelectrochemical water splitting materials†

**DOI:** 10.1039/d2sc05115j

**Published:** 2022-11-07

**Authors:** Ken J. Jenewein, Sigurd Thienhaus, Attila Kormányos, Alfred Ludwig, Serhiy Cherevko

**Affiliations:** Helmholtz-Institute Erlangen-Nürnberg for Renewable Energy (IEK-11), Forschungszentrum Jülich Cauerstrasse 1 D-91058 Erlangen Germany k.jenewein@fz-juelich.de s.cherevko@fz-juelich.de; Department of Chemical and Biological Engineering, Friedrich-Alexander-Universität Erlangen-Nürnberg Egerlandstrasse 3 91058 Erlangen Germany; Materials Discovery and Interfaces, Institute for Materials, Ruhr University Bochum Universitätsstraße 150 D-44801 Bochum Germany; Center for Interface-Dominated High Performance Materials, Ruhr University Bochum, Universitätsstraße 150 D-44801 Bochum Germany; Department of Physical Chemistry and Materials Science, Interdisciplinary Excellence Centre, University of Szeged Aradi Square 1 Szeged H-6720 Hungary

## Abstract

The experimental high-throughput (HT) exploration for a suitable solar water splitting photoanode has greatly relied on photoactivity as the sole descriptor to identify a promising region within the searched composition space. Although activity is essential, it is not sufficient for describing the overall performance and excludes other pertinent criteria for photoelectrochemical (PEC) water splitting. Photostability in the form of (photo)electrocatalyst dissolution must be tracked to illustrate the intricate relation between activity and stability for multinary photoelectrocatalysts. To access these two important metrics simultaneously, an automated PEC scanning flow cell coupled to an inductively coupled plasma mass spectrometer (PEC-ICP-MS) was used to study an Fe–Ti–W–O thin film materials library. The results reveal an interrelation between composition, photocurrent density, and element-specific dissolution. These structure–activity–stability correlations can be represented using data science tools like principal component analysis (PCA) in addition to common data visualization approaches. This study demonstrates the importance of addressing two of the most important catalyst metrics (activity and stability) in a rapid and parallel fashion during HT experiments to adequately discover high-performing compositions in the multidimensional search space.

## Abstract 2

Activity-stability relations of multinary oxide photoanodes for solar water splitting were measured using an automated flow cell coupled to an ICP-MS and successively visualized with the help of data science tools like principal component analysis.

## Graphical abstract

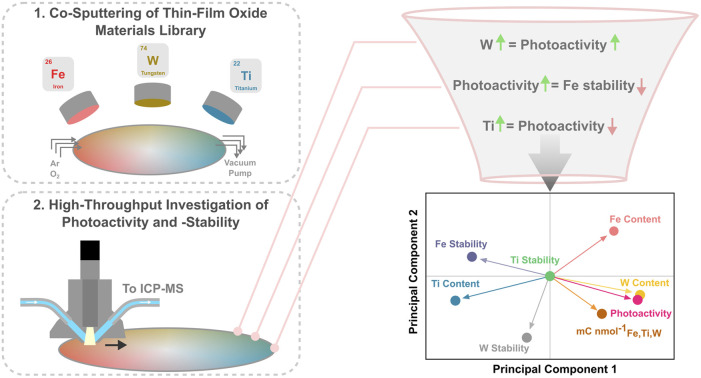

## Introduction

Advancing the industrial readiness of sustainable energy technologies is strongly tied to the availability of suitable electrocatalysts that meet stringent activity and stability requirements. The lack of an electrocatalyst that simultaneously possesses both of these attributes hampers the widespread application of several theoretically promising energy conversion schemes. Solar fuel production based on photoelectrochemistry (PEC) is one such research area that has been constrained by the absence of an ideal photoelectrocatalyst.^1^ The challenge lies in finding a candidate that is both a good photovoltaic and catalytic material. Such photoelectrocatalysts must meet a multitude of properties like (i) suitable band gap allowing large absorption of the solar spectrum; (ii) appropriate band edge position; (iii) high charge separation and transfer efficiency; (iv) earth abundancy; (v) high product selectivity; (vi) low toxicity; (vii) low production costs; and (viii) long term stability.^2,3^ Photoanodes must additionally overcome the sluggishness of the oxygen evolution reaction (OER), raising the bar even higher to find a potential all-rounder material. Generally, OER builds a cross-cutting platform to realize promising electrochemical transformations of H_2_O, CO_2_, or N_2_ to value-added hydrogen, C-containing, or N-containing fuels, respectively. Oxides are among the most promising material classes for photoanodes since they possess several of the aforementioned properties while forming relatively stable compounds. The high electronegativity of atomic oxygen results in the formation of oxidatively stable compounds based on O^2−^.^4^ However, simple binary oxides (one element forming a compound with oxygen) are highly unlikely to fulfill the tough requirements for PEC OER. In fact, almost all possible binary oxides have been explored already with only limited success.^5–7^ Thus, venturing into more complex multinary oxide systems presents a major opportunity to balance the deleterious properties of one material with the beneficial characteristics of another.^7^ The combinatorial possibilities offered by ternary (>19 000) or quaternary (>220 000) oxides, considering about 50 possible candidate metals in the periodic table, give a high dimensional search space from which the chances to identify a candidate with all desired properties of an ideal photoanode material are higher.^5,8^ Many ternary systems have been explored in the past, but the inherent limitations of charge transfer and separation efficiency, PEC kinetics, and durability still persist.^6,9^ Quaternary oxides are just recently gaining interest and several reports have already demonstrated viable combinations for PEC water splitting.^10–13^ An interesting quaternary oxide is the system Fe–Ti–W–O.^14^ Binary oxides such as Fe_2_O_3_,^15^ TiO_2_,^16^ and WO_3_ (ref. 17) are all well-studied representatives for photoanode materials, and investigating their combination for potential synergistic effects is therefore logical. Ternary systems based on two of the three transition metals have also been studied in the past, including the compounds Fe_2_TiO_5_,^18–20^ Fe_2_WO_6_,^21^ as well as materials libraries in the system Fe–W–O^22^ and Ti–W–O.^23^ While Fe_2_WO_6_ is claimed to be less viable as a photoabsorber, Fe_2_TiO_5_ was found to exhibit superior surface charge transfer efficiencies compared to Fe_2_O_3_, and a heterojunction out of Fe_2_TiO_5_/Fe_2_O_3_ outperformed the photocurrent of just Fe_2_O_3_ by an order of magnitude.^18^

With the increasing combinatorial diversity of multinary systems, high-throughput (HT) screening methods must concomitantly evolve to investigate the vast number of compositions in an accelerated manner.^24–27^ The underlying premise of most HT assessments is to identify hit regions indicated by compositions with the highest activity. Although the related OER kinetics are a large consideration when assessing the suitability of a photoanode, it does not provide insights into other characteristics critical to use in real conditions. Thus it is important to precisely define extended criteria for promising hit candidates within the search space.^5^ Assessing additional parameters within the HT workflow that would paint a more complete picture of the photoanode performance must be balanced with a reasonable screening time for a composition space.^28^ Only a photoanode with a high oxygen turnover frequency and exceptional operational durability can be classified as a high-performing and desirable material for PEC applications, making photostability a key parameter to track during HT explorations of interesting systems.^29^ HT photostability screening of material libraries has been realized in the past by examining the decay in photocurrent or qualitatively by observing whether measured areas vanished after PEC operation.^30,31^ This approach allows the rapid assessment of the overall performance retention but only gives a first glimpse into the material stability. It remains elusive whether the performance drop is related to factors such as phase transformation, catalyst restructuring, or corrosion in the form of catalyst leaching of one or several elements. Real-time corrosion measurements can be a powerful toolkit for gaining detailed insights into degradation mechanisms during photoelectrochemical operations.^32–35^ This method has been realized through the coupling of a photoelectrochemical scanning flow cell to a highly sensitive inductively coupled plasma mass spectrometer (PEC-ICP-MS). Employing an ICP-MS would allow for the detection of trace amounts of dissolved species during HT activity measurements.^28,32,35,36^ In fact, ICP-MS is often used to measure dissolved metal concentrations in (photo)electrochemical applications during long-term measurement.^37,38^ Such complementary analyses are critically needed to advance (photo)electrocatalysts and extend the discussion on OER performance beyond ordinary metrics like activity.^39^ The simultaneous evaluation of the activity–stability relation within one HT screening cycle eliminates the need for extended PEC protocols to evaluate the performance drop of each sample and can further inform and complement post-mortem analysis. Automating PEC-ICP-MS measurements can bring a new dimension to HT endeavors in which the stability can be mapped in the form of dissolved ions for an entire material library. HT on-line ICP-MS methods were recently combined with machine learning approaches to accelerate the development of molten salt corrosion-resistant alloys, demonstrating the range of studies that this technique can address.^40^

In this study, a Fe–Ti–W–O material library fabricated by combinatorial reactive co-sputtering was examined for its photoactivity and photostability simultaneously using an automated PEC-ICP-MS setup. Based on the synchronously obtained photocurrent density and amount of each dissolved metal, it is possible to construct a metric joining both contributions, which can then be projected over the composition space. Tools from the data science community are a great asset to HT experiments by facilitating the interpretation of complex, high-dimensional data. In this line, principal component analysis (PCA) was employed to perform a dimensionality reduction. Adapting the PEC-ICP-MS method for HT experiments can bring significant advancements to the accelerated exploration of undiscovered high-performing photoanodes with respect to two crucial catalyst metrics.

## Results and discussion

### High-throughput photoactivity screening

**Fig. 1:**
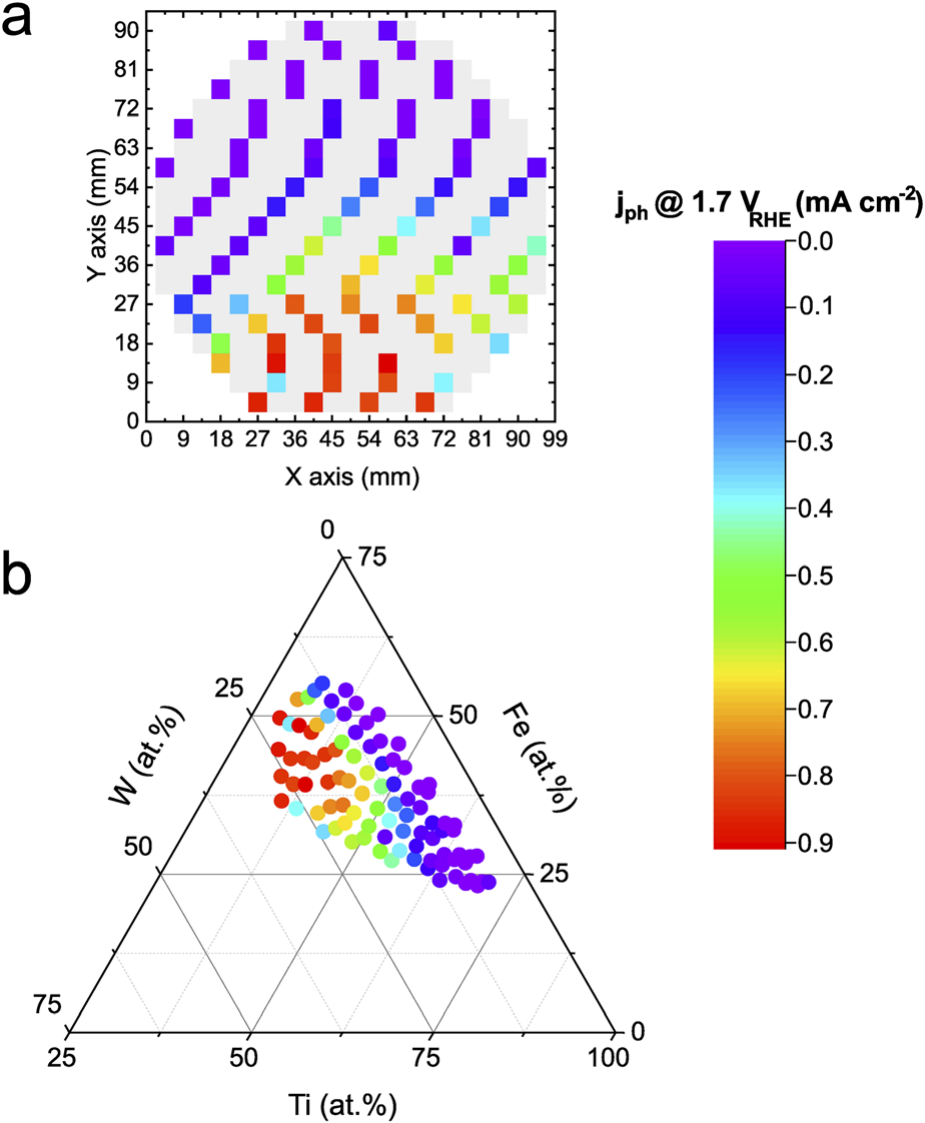
Photocurrent densities of Fe–Ti–W–O material library at 1.7 V_RHE_. (a) Photocurrent density mapped onto a schematic representation of the wafer. (b) Photocurrent density mapped onto a ternary composition diagram of the metal constituents. The HT measurements were conducted in 0.05 M NaNO_3_ (pH 5.7) under 1 Sun Am 1.5G illumination.

The transient photocurrent density was measured at 1.7 V_RHE_ in a near-neutral electrolyte (pH 5.7). Fe is thermodynamically unstable under acidic conditions, whereas W destabilizes under alkaline pHs. Hence a near-neutral pH was intentionally chosen to investigate the Fe–Ti–W–O material library in conditions that should a *priori* not destabilize any of the components directly. Fig. 1a shows a color-coded schematic representation of the measured Fe–Ti–W–O materials library with the photocurrent densities mapped on top. Grey tiles indicate measurement areas (MAs) where the photocurrent density was not determined. Excluding some MAs from measurements assured faster screening of the whole library without losing too much information about persistent trends. This way, the HT activity–stability screening can be performed within 4–5 h, corresponding to an acceleration factor of about 100× compared to classical screening methods, where each sample is measured manually, for example, in a bulk cell. Automating *in situ* dissolution measurements significantly contribute to this high acceleration factor, since traditional ICP-MS measurements are typically slow and labor-intensive.

The mapped photocurrent densities visualize the activity distribution on the wafer geometry of the materials library, but the actual composition dependence cannot be derived. Therefore, the obtained activity results must be unified with the corresponding elemental composition obtained from HT energy dispersive X-ray (EDX) measurements (see Fig. S1†), as depicted in Fig. 1b. The merged data is illustrated in a ternary diagram showing that a higher W and Fe content increases the photoactivity. For Ti contents above 60 at%, photocurrent density values decrease significantly, which is attributed to the low visible light absorption characteristic of TiO_2_. The overall trend is congruent with the literature.^14^

### High-throughput photostability screening

**Fig. 2:**
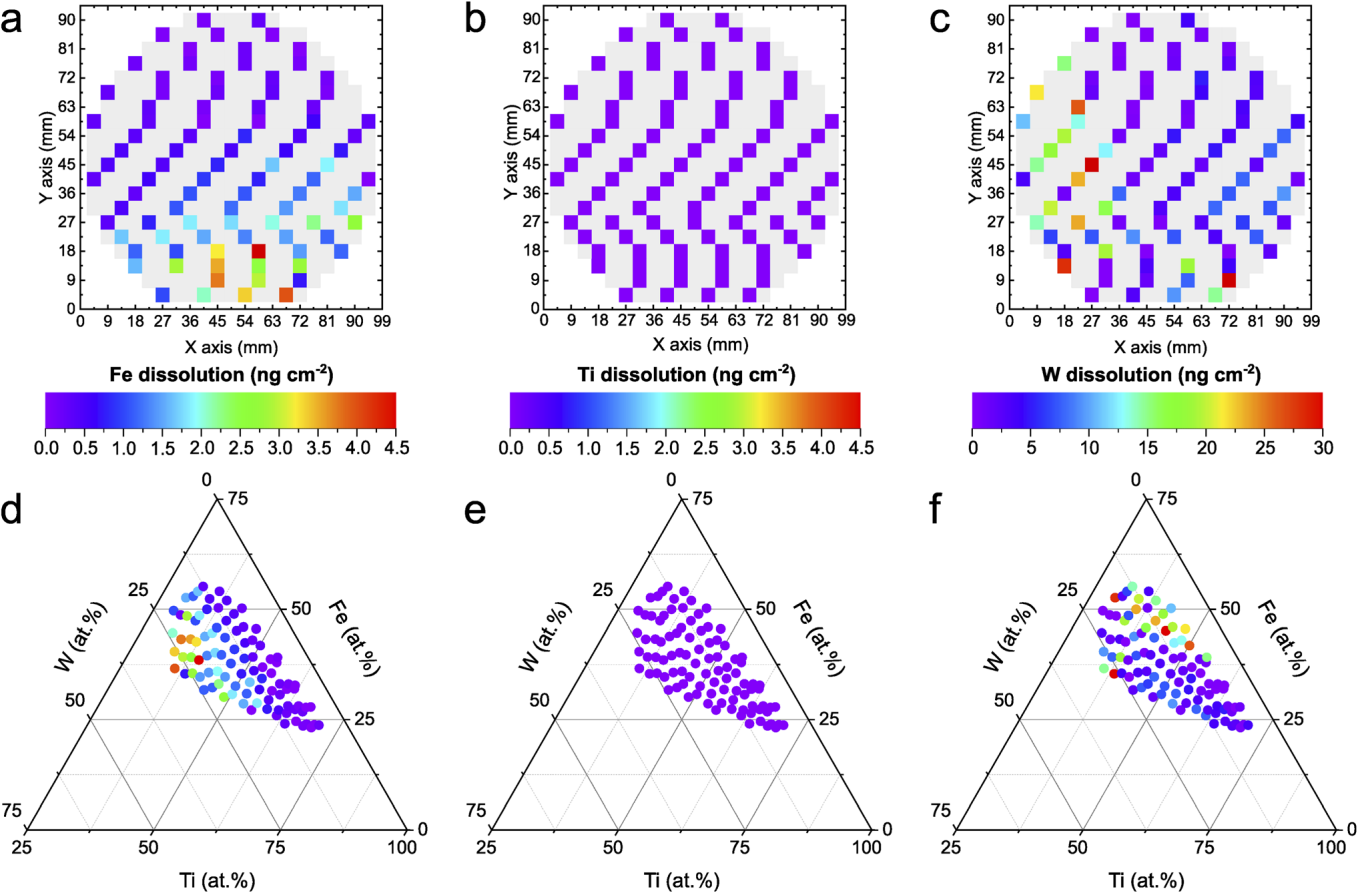
Element-specific dissolution of Fe–Ti–W–O material library at 1.7 V_RHE_. (a–c) Dissolved amounts of Fe (a), Ti (b), and W (c) mapped onto a schematic representation of the wafer. (d–f) Dissolved amounts of Fe (d), Ti (e), and W (f) mapped onto a ternary diagram. The HT measurements were conducted in 0.05 M NaNO_3_ (pH 5.7) under 1 Sun Am 1.5G illumination. Note that the color scale for W differs from Fe and Ti.

The automated PEC-ICP-MS method tracks the amount of dissolved metals during the HT photoactivity screening shown in Fig. 1. Less metal leaching is associated with higher stability. The elemental resolution of the dissolution signal allows for identifying the existence of any preferential dissolution of a specific element. As shown in Fig. 2a–c, W shows almost one order of magnitude higher dissolution than Fe. On the other hand, Ti dissolves in amounts below the detection limit of the instrument (1 ppb), if at all. As discussed earlier, the composition-dependent trend is scientifically more insightful. The dissolved amounts for each measured composition of the Fe–Ti–W–O system are mapped onto a ternary diagram, as shown in Fig. 2d–f. Fe dissolution (Fig. 2d) increases for photoanodes with relatively higher W content and shows a similar trend to the photoactivity in Fig. 1. This can be explained by the fact that Fe is more sensitive to local pH changes during PEC OER compared to the other constituents. The acidification caused by proton generation during OER can destabilize Fe significantly.^41^ Such local pH changes could be mitigated by, for instance, employing buffered electrolytes that neutralize generated protons immediately. The increasing Fe dissolution at higher photocurrent densities might suggest that a significant portion of the photocurrent is originating from Fe oxidation currents. To illustrate the insignificance of the oxidative Fe degradation to the total photocurrent density, the composition Fe_39_Ti_39_W_22_O_*x*_, where the Fe dissolution was the highest (4.5 ng cm^−2^), was used for an example calculation. In this case, just 0.35% of the entire oxidative charge can be related to Fe dissolution under the assumption of the following photocorrosion process:^42^

1 Fe_2_O_3_ + 6h^+^ ↔ 2Fe^3+^ + 1.5O_2_

The high stability of Ti in neutral pH (Fig. 2e) is expected, as corresponding Pourbaix diagrams indicate a stable region in such conditions.^43^ Interestingly, W dissolution exhibits a nearly opposite trend compared to the one shown for Fe, in which W dissolution increases with less W and more Fe within the compositions. While the exact reason for this observation remains the subject of further detailed investigations, it was shown that the sputtered film of the Fe–Ti–W–O library consists of multiple phases (hexagonal WO_3_, trigonal FeTiO_3_, orthorhombic Fe_2_TiO_5_, and tetragonal Ti_0.54_W_0.46_O_2_).^14^ A differing phase distribution in relation to the composition could trigger a possible phase-dependent destabilization.

### Joint photoactivity and photostability metric

HT explorations search for a hit region based on suitable criteria defining a promising photoanode material. Photoactivity alone is not sufficient and important intrinsic properties such as photostability must be tracked simultaneously. A joint metric is needed to facilitate the interpretation of the recorded activity and stability data. In the past, the stability number (S-number) was proposed, allowing the comparison of OER electrocatalysts with respect to their activity and stability.^44^ The S-number was later modified to account for any oxidative reactions where a 100% faradaic efficiency for OER is not guaranteed.^33^ Originally, this metric only considered a single element, even when a catalyst with multiple constituents is assessed.^45^ As there is no consensus on how to combine the contribution of all dissolved elements in a multi-elemental system, we propose a new approach that normalizes the photocharge with the total dissolved mols of metal according to:2
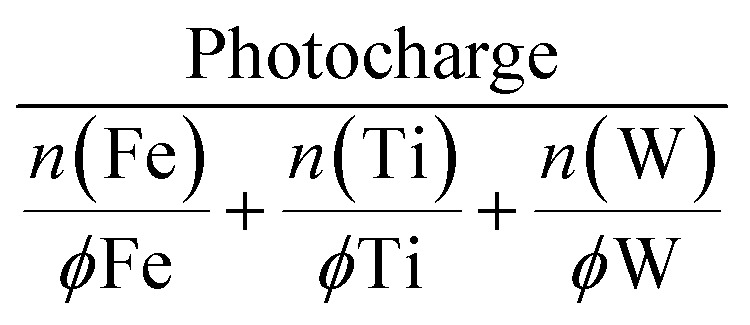


The photocharge was extracted from the integral of the photocurrent signal. The dissolved mols of each metal (*n*) were first normalized by the fractional amount (*ϕ*) present within the measured composition. This normalization accounts for the simple linear scaling between the metal content in the material library and the respective metal loss. This way, the severity of degradation can be accurately compared across samples with different compositions. For example, a metal dissolution of 2 nmol has a different implication on the degree of degradation when the initial metal content is 10 or 1000 nmol.

**Fig. 3:**
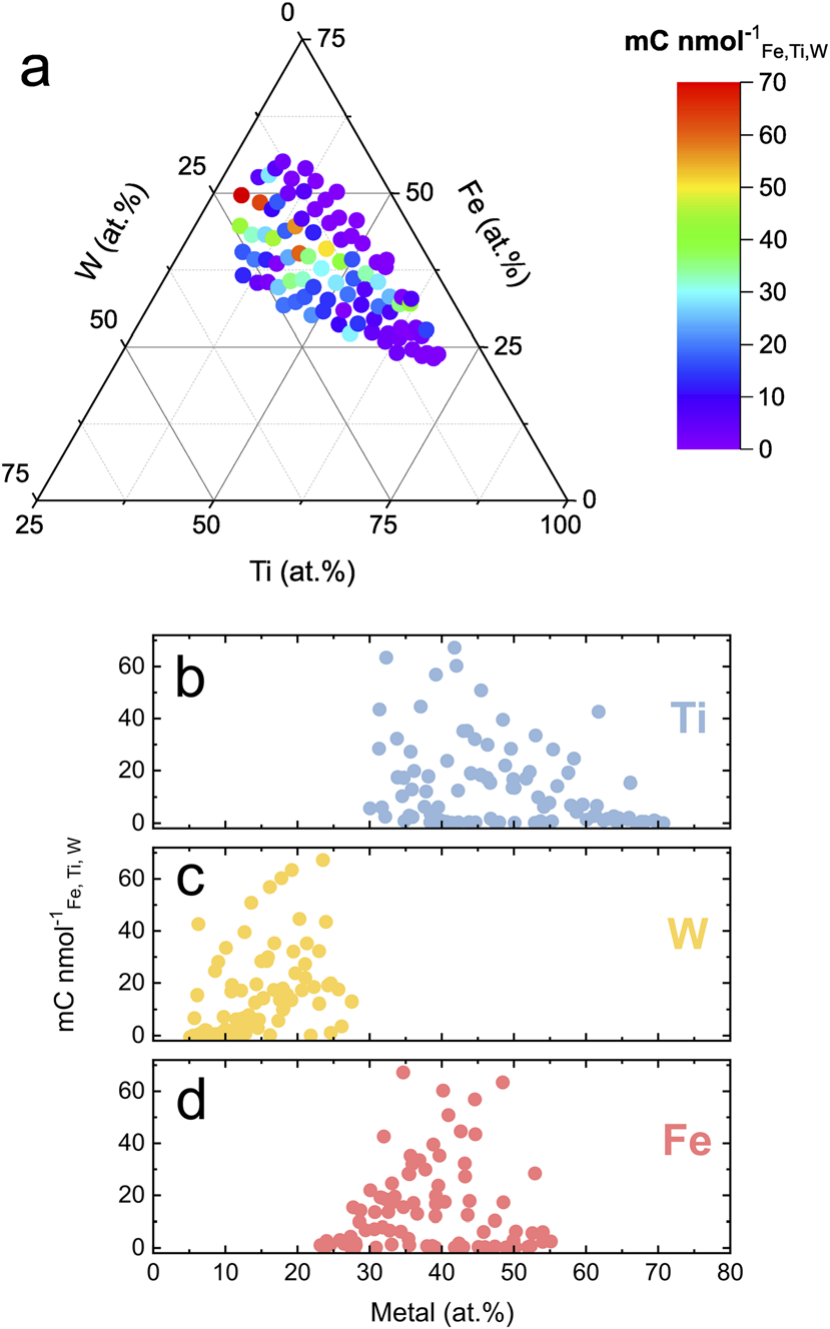
Photocharge normalized by the total dissolution of the Fe–Ti–W–O material library measured at 1.7 V_RHE_. (a) Dissolution-normalized photocharge mapped onto a ternary diagram. The performance metric is further plotted over the specific Ti (b), W (c), and Fe (d) content in the oxide films.

Fig. 3a shows the dissolution normalized photocharge and hints toward a highly active and stable region around Fe_43_Ti_36_W_21_O_*x*_. This composition fits within the Fe_30–49_Ti_29–55_W_13–22_O_*x*_ hit region previously identified for this quaternary oxide system based on photoactivity.^14^ Moderately performing compositions stretch into lower W contents when keeping the Fe fraction around 35 at%. The activity–stability metric can be further deconvoluted by replacing the denominator with the dissolution of an individual element rather than the total dissolution. The corresponding ternary diagrams are presented in Fig. S2,† where the Ti-normalized photocharge has been omitted since the division by zero (no Ti dissolution) is mathematically impossible. This deconvolution reveals that the low-performing compositions in Fig. 3a are majorly due to their low W content.

To visualize trends encountered in Fig. 3a from a different perspective, the dissolution normalized photocharge was plotted over the specific fraction of each element (Fig. 3b–d). The performance of the compositions from the Fe–Ti–W–O materials library becomes poorer when either the Ti content increases above 60 at% or the W content decreases below 20 at%. The joint activity–stability metric does not show a clear dependency on Fe content.

### Extracting relations with principal component analysis

Ternary diagrams are useful to illustrate four-dimensional data (one measured property for each oxide composition), but with increasing throughput and complexity of HT campaigns, data sets derived from such experiments become high-dimensional and less readily comprehensible. Proper data visualization is crucial as interpretability becomes increasingly challenging. Simplifying the data as attempted in Fig. 3b–d quickly approaches a limit on the number of parameters that can be clearly visualized in parallel and is inadequate to display interrelations between several measured variables. Tools applied in data science can help to represent intricate underlying correlations in multivariate data. For instance, plotting the pairwise correlation coefficients in a heatmap, as shown in Fig. S3,† is one strategy. However, this representation can become crowded with increasing numbers of variables. Another technique that can be helpful is the so-called principal component analysis (PCA). Here, complex data can be projected in an easily visualizable space by reducing the dimensionality.^46^ The elemental composition (Fe, Ti, and W), photocurrent density, element-specific dissolution, and the dissolution-normalized photocharge were used as inputs for the dimensionality reduction. The dissolution values were negated to imply a beneficial effect on the photoanode of all property variables with higher values. Therefore, the term “dissolution” is replaced by “stability” in the upcoming PCA discussion. All the data were centered and scaled previous to the PCA to prevent assigning a higher weight to variables with larger numeric values, which would skew the PCA outcome.

**Fig. 4:**
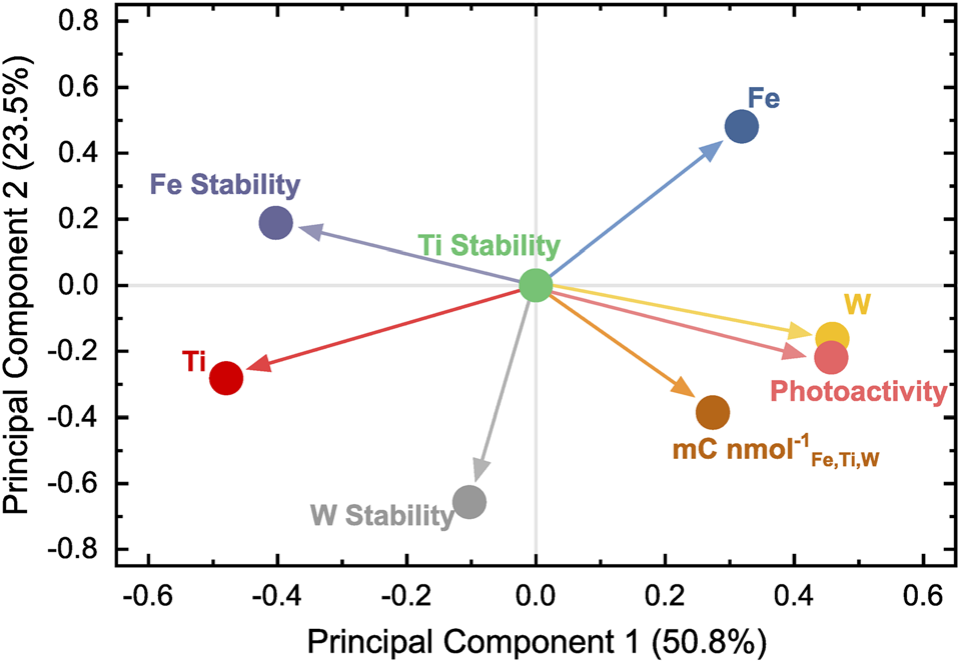
Loading plot for the first two principal components obtained by PCA.

Fig. 4 shows the results of the PCA in the form of a loading plot. The first two principal components explain 74.3% of the variance in the data set. The corresponding scree plot is shown in Fig. S4.† A loading plot helps to deduce the effect of chemical composition on the photoanode performance represented as activity, stability, and the joint activity–stability metric. Clustering of loading points close to each other means the variables show a positive correlation. On the other hand, loading points separated by the origin have a negative relation.

Photoactivity appears to have an inverse effect on the Fe stability, meaning higher photocurrents are correlated to the destabilization of Fe. The performances of photoanode compositions are most strongly correlated with the overall W content. Ti seems to be beneficial for the photoanode stability since the loading point for Ti is located closely to all stability variables. The low dissolution with high Ti content comes at a price, namely low activity, indicated by the photoactivity and Ti being positioned on the opposite sides of the loading plot. The PCA expresses the trends already observed visually during the HT campaign and proves its capability to represent various trends in a simple graph.

## Experimental

### Fabrication of Fe–Ti–W–O thin film material libraries

The synthesis procedure was elaborated in detail elsewhere.^14^ In short, metallic targets of Fe (99.95%), Ti (99.95%), and W (99.99%) were used for the reactive co-sputter deposition on a 4-inch diameter Si/SiO_2_ wafer substrate. To enable PEC measurements, a 100 nm thick Pt conducting back electrode layer was sputtered onto the Si/SiO_2_, coated before with a 10 nm thick Ti adhesion layer. The reactive gas was a mixture of Ar (30 sccm) and O_2_ (3 sccm) at a deposition pressure of 0.66 Pa. W and Ti were sputtered using direct current (DC) while Fe was sputtered by using a radio frequency (RF) power supply. The as-deposited thin-film libraries were annealed in ambient air at 500 °C for 8 h. The temperature ramp rate was set to 15 °C min^−1^, followed by natural cool down.

### High-throughput composition characterization

The elemental compositions of each MA of the materials library were determined using automated energy dispersive X-ray (EDX, INCA X-act detector, Oxford instruments) analysis in a scanning electron microscope (SEM, JEOL 5800) performed at an acceleration voltage of 20 kV in the center of each MA.

### High-throughput PEC-ICP-MS measurements

A detailed overview of the PEC-ICP-MS setup can be found elsewhere.^32^ Light was generated from a 300 W ozone-free Xe lamp (Newport), which was passed through an AM 1.5G filter (Newport) before getting channeled into the photoelectrochemical scanning flow cell (PEC-SFC) *via* a liquid light guide (Newport). The light was calibrated at the cell opening to 1 Sun (100 mW cm^−2^) using a reference solar cell (Newport).

The three-electrode configuration on the PEC-SFC was formed by using a Ag/AgCl electrode in 3 M KCl (Metrohm) as the reference, a glassy carbon rod (SIGRADUR G, HTW) as the counter electrode, and the sample as working electrode. PEC protocols were controlled with a Gamry Ref 600 potentiostat. All experiments were performed with 0.05 M NaNO_3_ (99.99%, Sigma, pH 5.7) as supporting electrolyte. The PEC protocol consisted of a 20 s hold at 1.7 V_RHE_ during which the first 10 s were illuminated and the last 10 s were performed in dark. The photocurrent density was calculated by averaging the last 20% of the data gathered during the illuminated period and subtracting the background current density recorded during the dark cycle.

The first measurement area (6.25 mm to the left and 6.75 mm to the top from the left flat wafer edge) was contacted manually with the PEC-SFC with a force of 0.6 N. From here, an automated sequence is executed in the following order: (i) the PEC protocol is executed. (ii) After the measurements, the substrate is decontacted by 200 μm. (iii) The stage moves in the *X*–*Y* plane to reach the new measurement area while dragging the continuously flowing meniscus over the substrate. Once the translation in the *X*–*Y* plane is completed, the PEC-SFC is contacted with the substrate again. The iteration then starts over at (i).

*In situ* dissolution signals obtained from the ICP-MS were quantified using a four-point calibration curve (0, 0.5, 1, 5 μg L^−1^) for Fe, Ti, and W. ^59^Co (for ^56^Fe), ^45^Sc (for ^48^Ti), and ^187^Re (for ^184^W) at a concentration of 50 μg L^−1^ served as internal standard. The ICP-MS was operated under the dynamic reaction cell mode using methane as cell gas at a flow rate of 0.3 mL min^−1^. This ensured the elimination of ^40^Ar^16^O^+^ polyatomic species generated by the plasma interfering with ^56^Fe.

### Principal component analysis

PCA is a multivariate method of converting a large number of variables into new mathematical variables called principal components. Elemental fractions (Fe at%, Ti at%, and W at%), dissolved amounts (for Fe, Ti, and W), photocurrent density, and the dissolution normalized photocharge were all used as variables for the PCA. All numerical values were centered and scaled previously to the dimensionality reduction so that each data axis has a zero mean and unit variance. Python was used to conduct appropriate data treatment with the Pandas^47^ and Numpy^48^ packages. The actual PCA was done using the Scikit-learn^49^ package.

## Conclusions

In summary, this work has demonstrated the applicability of an automated PEC-ICP-MS device to successfully reveal activity–stability relations in a Fe–Ti–W–O photoanode library for PEC water splitting. The highest performance considering the photoactivity and stability was observed around compositions of Fe_43_Ti_36_W_21_O_*x*_. While Ti did not show any signs of leaching, Fe dissolution showed a similar compositional trend as the photocurrent. W dissolved an order of magnitude more than Fe and displayed a nearly inverse trend to the photoactivity. Results from PCA show that both the activity and the overall performance, represented as a joint activity–stability metric, correlate positively with the W content in the Fe–Ti–W–O thin-film library. Higher Ti contents seem to have a stabilizing effect but lead to a dramatic performance drop instead. This study emphasizes the importance of photostability during HT explorations for novel multinary photoanode compositions and illustrates the feasibility of open-source data treatment tools to interpret high-dimensional data sets.

## Data availability

All data presented in this work can be found at https://github.com/kjenewein/High-Throughput-PEC-ICP-MS. The same link contains a python code used to perform the principal component analysis.

## Author contributions

K. J. J. designed and performed all PEC-ICP-MS procedures and the evaluation of the obtained data. S. T. provided the Fe–Ti–W–O material libraries. K. J. J. wrote the original draft. S. T., A. K., A. L., and S. C. reviewed and edited the manuscript. A. K. and S. C. supervised the experimental work.

## Conflicts of interest

There are no conflicts to declare.

## Supplementary Material

SC-013-D2SC05115J-s001
