# Supplementary material for: High-throughput exploration of activity and stability for identifying photoelectrochemical water splitting materials
Source: Chem Sci. 2022 Nov 7;13(46):13774–81. doi: 10.1039/d2sc05115j (PMC9710305; doi:10.1039/d2sc05115j)
Supplement: SC-013-D2SC05115J-s001 [file SC-013-D2SC05115J-s001.pdf]

Electronic Supplementary Material (ESI) for Chemical Science.
This journal is © The Royal Society of Chemistry 2022

Supplementary Information

High-throughput exploration of activity and stability for identifying photoelectrochemical water splitting materials

Ken J. Jenewein,[1,2]* Sigurd Thienhaus,[3,4] Attila Kormányos,[1,5] Alfred Ludwig,[3,4] and Serhiy Cherevko[1]*

[1] Helmholtz-Institute Erlangen-Nürnberg for Renewable Energy (IEK-11), Forschungszentrum Jülich, Cauerstrasse 1, 91058 Erlangen, Germany

[2] Department of Chemical and Biological Engineering, Friedrich-Alexander-Universität Erlangen-Nürnberg, Egerlandstrasse 3, 91058 Erlangen, Germany

[3] Materials Discovery and Interfaces, Institute for Materials, Ruhr University Bochum, Universitätsstraße 150, D-44801 Bochum, Germany

[4] Center for Interface-Dominated High Performance Materials, Ruhr University Bochum, Universitätsstraße 150, D-44801 Bochum, Germany.

[5] Department of Physical Chemistry and Materials Science, Interdisciplinary Excellence Centre, University of Szeged, Aradi Square 1, Szeged, H-6720, Hungary

*Corresponding author k.jenewein@fz-juelich.de s.cherevko@fz-juelich.de

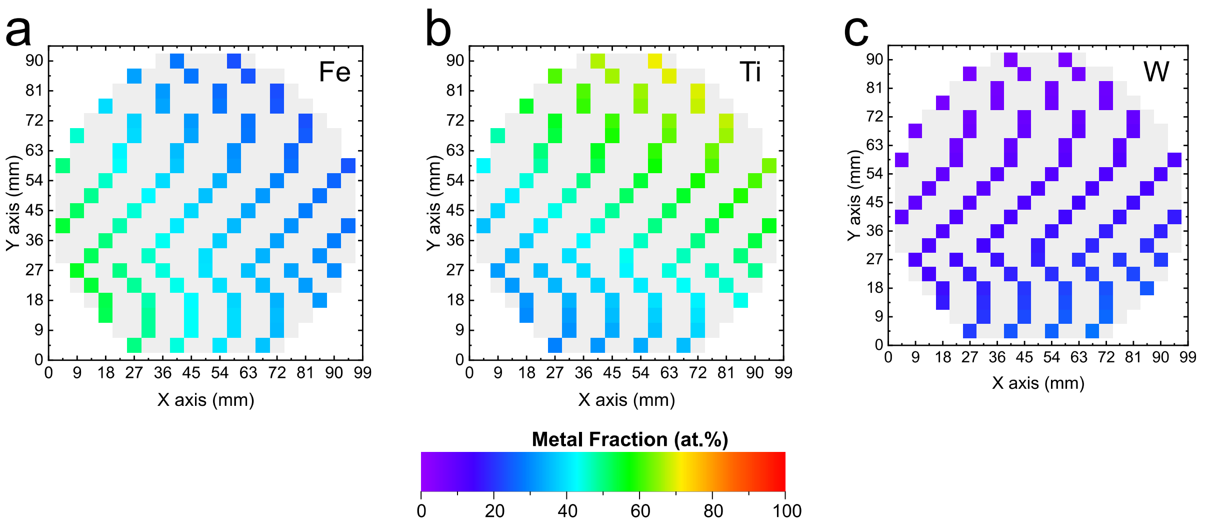

Figure S1: HT EDX elemental mapping of the Fe-Ti-W-O material library. (a) Fe content. (b) Ti content. (c) W content. Gray tiles represent areas within the wafer that were not subjected to the HT activity-stability screening.

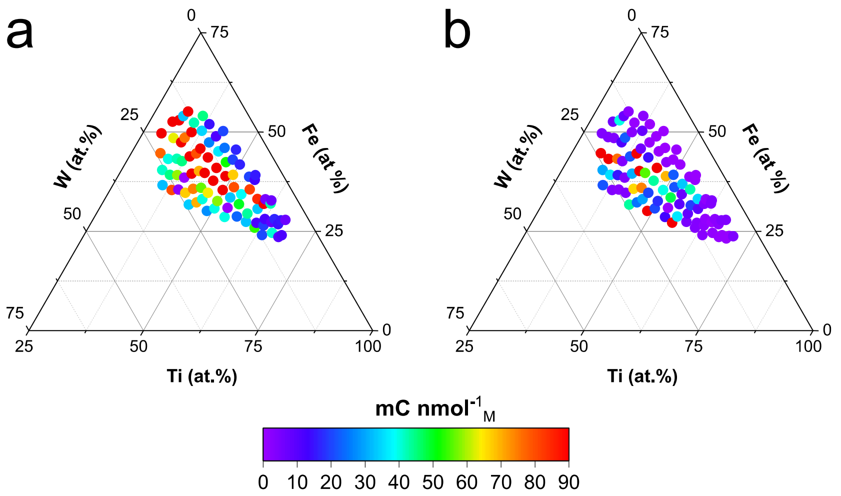

Figure S2: Deconvoluted dissolution-normalized photocharge. (a) Photocharge normalized by fraction-corrected Fe dissolution. (b) Photocharge normalized by fraction-corrected W dissolution.

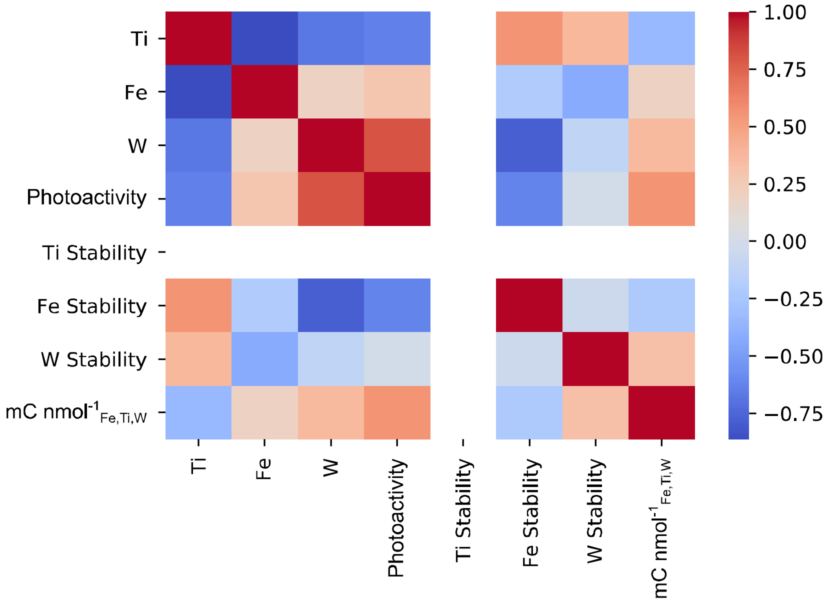

Figure S3: Heatmap showing the pairwise correlation of each variable studied in the present work

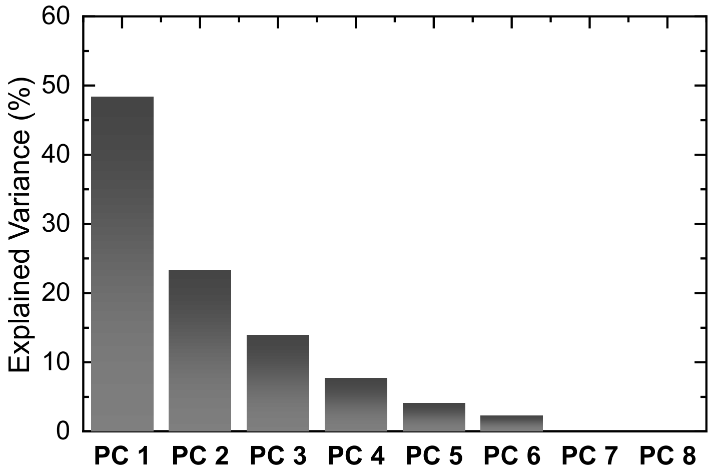

Figure S4: Scree plot for PCA
